# Genetics and Epigenetics in Acquired Hemophilia A: From Bench to Bedside

**DOI:** 10.3390/cimb46060309

**Published:** 2024-05-23

**Authors:** Nikolaos Evangelidis, Nikolaos Kotsiou, Paschalis Evangelidis, Vlasios I. Alevizopoulos, Iasonas Dermitzakis, Sofia Chissan, Sofia Vakalopoulou, Eleni Gavriilaki

**Affiliations:** 1 Hematology Unit-Hemophilia Centre, 2nd Propedeutic Department of Internal Medicine, Hippocration Hospital, Aristotle University of Thessaloniki, 54642 Thessaloniki, Greece; evangeln@auth.gr (N.E.); kotsiounikolaos@gmail.com (N.K.); pascevan@auth.gr (P.E.); vlasiosa@auth.gr (V.I.A.); sofiachissan@yahoo.com (S.C.); svakalopoulou@yahoo.com (S.V.); 2 Medical School, Faculty of Health Sciences, Aristotle University of Thessaloniki, 54124 Thessaloniki, Greece; iasonasd@auth.gr

**Keywords:** acquired hemophilia, bleeding disorders, epigenetics, genetics, hematology

## Abstract

Acquired hemophilia A (AHA) is a bleeding disorder characterized by the immunological inhibition of factor VIII (FVIII) of the hemostatic pathway leading to hemorrhagic events. Different domains of FVIII are the target of autoantibodies (mainly immunoglobulin (Ig) G) leading to the deficiency of FVIII. Several factors have been associated with the activation of the auto-immunity towards FVIII. Emerging evidence implicates CD4+ T cell activation in mediating this autoimmune response, with their involvement like that observed in congenital hemophilia A. Several genes such as HLA II DRB*16, DQB1*0502, and CTLA-4 + 49 are responsible for the pathogenesis of AHA. Epigenetic modifications and mainly long-coding RNAS (lncRNAs) are potentially contributing to the pathogenesis of AHA. The treatment approach of AHA includes the management of acute bleeding events and the administration of immunosuppressive medications. This review aimed to summarize the published data on the genetics and epigenetics of AHA. The severity and the mortality of this disease are creating an emerging need for further research in the field of the genetics and epigenetics of acquired hemorrhagic disorder.

## 1. Introduction

Hemophilia is a hemostatic disorder resulting in hemorrhagic incidents in patients. Two congenital types of hemophilia are the most common, hemophilia A (HA) (1 in 5000 male births), in which there is a lack or deficiency of factor VIII (FVIII), and hemophilia B (HB) (1 in 30,000 births), accompanied by a lack or deficiency of factor IX (FIX) [1,2]. The genes that code FVIII and FIX are F8 and F9 accordingly in the X chromosome; thus, HA and HB are X-linked disorders and are inherited by X-linked recessive patterns [3]. The severity of the disease depends on the decrease in the FVIII or FIX levels [4]. Replacement therapy, with the administration of the missing factor, remains a cornerstone in the management of congenital hemophilia, to treat both bleeding and prophylactically [5]. Patients with congenital HA and HB have a variety of therapeutic options, including gene and cellular therapies [6,7]. Progressive joint disease, the development of inhibitors, increased risk of cardiovascular disease (CVD), infections, and severe bleeding constitute complications of these clinical entities, leading to increased mortality and morbidity [8,9,10,11,12]. Next-generation therapeutics have been introduced in everyday clinical practice for the management of congenital hemophilia [13]. Next-generation therapeutics include the development of the gene therapy of congenital HA and recombinant antihemophilic factors [14,15].

Acquired hemophilia A (AHA) is a rare, potentially life-threatening, acquired bleeding disorder [16]. The cause of AHA is the development of neutralizing autoantibodies (inhibitors) against FVIII. The incidence is about 1.5 cases per million people every year [17]. AHA mainly affects adults over 65 years old, while it has been observed in young females between 20 and 30 years during pregnancy or in the postpartum period [18,19]. Fifty percent of AHA cases are considered idiopathic because an underlying cause is not recognized [18]. The rest of these cases have been attributed to autoimmune disorders (such as systemic lupus erythematosus and rheumatoid arthritis), hematological malignancies (such as b-lymphoproliferative disorders), solid tumors, infections, drugs, and pregnancy [18,20,21,22]. Moreover, AHA is characterized by the sudden onset of bleeding, spontaneously or after an invasive procedure, in a patient without a personal or family history of bleeding [23]. The laboratory confirmation of AHA is established by low FVIII levels and the detection of an anti-FVIII antibody, quantified in Bethesda units (BUs) [24]. It is underlined that the severity of bleeding is not associated with FVIII level or inhibitor titer detected at the time of diagnosis (baseline) [25].

Immune dysregulation is a key step in the pathogenesis of AHA [26]. The aim of the current literature review is to investigate the genetic and epigenetic base of AHA. Thus, a literature search was performed on the PubMed database using the following keywords: “acquired hemophilia”, “AHA”, “acquired bleeding disorder”, “immune system”, “genetic”, “gene”, “epigenetics”, “microRNAs”, and “treatment”, in different combinations. To our knowledge, this is one of the first reviews examining the molecular biology of this complex clinical entity. Moreover, we summarize the basic principles in the management of patients with AHA. In the era of personalized medicine, a better understanding of AHA pathogenesis is crucial. The interplay between immunity, genetics, and epigenetics and improved knowledge of these mechanisms is essential for the development of targeted and personalized therapies for the treatment of AHA and other diseases following this pattern.

## 2. Immune System and Acquired Hemophilia A

FVIII is a glycoprotein that circulates in the bloodstream and is produced in endothelial cells (mainly in the sinusoidal endothelial cells of the liver) [27]. The structure of the FVIII protein is as follows: A1, A2, B, A3 C1, and C2 domains and acidic spacers [28]. The A1–A2–B domains form the heavy chain, while A3–C1–C2 form the light chain [29]. Heavy and light chains are complexed to von Willebrand Factor (vWf) until the activation of FVIII by thrombin, where the B domain, the acidic a3 region, and the von Willebrand dissociate the FVIIIa [30]. FVIIIa (on the activated platelets membranes), phospholipid membrane, and FIXa will form the tenase complex that activates factor X [31]. AHA is considered to be an autoimmune disorder in which autoantibodies are generated against molecules that are related to endogenous FVIII or against endogenous FVIII itself, causing the neutralization of its action [24].

### 2.1. Types of Autoantibodies Produced in AHA

The measurement of the autoantibodies can be implemented via an ELISA or Bethesda assay (Nijmegen modification). Neither Bethesda units nor residual FVIII activity can predetermine the bleeding phenotype of the patient. Thus, recognition of the type of autoantibodies produced in AHA is crucial for the clinical administration of the patient with AHA [32,33].

Low titers of autoantibodies against FVIII can be found in the general population too, approximately in 15–19% without any clinical manifestation [34]. Most autoantibodies are IgG1 and IgG2 subtypes mainly against the C2 domain of FVIII. The hemostatic phenomena are not present in this healthy group because of the production of anti-idiotype antibodies that neutralize circulating autoantibodies against FVIII [34]. Whether these non-neutralizing IgG antibodies in non-hemophilic patients predispose a person to develop acquired hemophilia or not is unclear [34,35,36].

The spectrum of the autoantibodies that can be found in acquired hemophilia involves a polyclonal population of IgG (mainly IgG1 and IgG4) but also IgA and IgM autoantibodies at lower percentages and titers [34]. IgA and IgM isotypes have been observed in patients with lymphoid neoplasms and paraproteinemias [37,38]. Different cohorts of patients investigating the type of antibodies observed in postpartum women with AHA concluded that IgG1 and IgG3 autoantibodies are mostly observed [39,40]. The study by Reding et al. showed that more intense anti-FVIII antibody activity and higher inhibitor titers meet with a predominance of Th2-driven IgG4 [41]. The GTH-AH 01/2010 study presented that the anti-FVIII IgG autoantibody is highly sensitive, 0.99 (95% confidence interval [CI], 0.59–1.0), and specific, 0.83 (95% CI, 0.72–0.91), for diagnosing acquired hemophilia, while its concentrations may have prognostic value, predicting a lower rate of remission and a higher possibility of death [42]. The same study concluded that IgA anti-FVIIIA is connected with an increased risk of relapse after therapy (adjusted hazard ratio (aHR), 0.35; 95% CI, 0.18–0.68; *p* < 0.01) and increased danger of death from the disease (aHR, 2.62; 95% CI, 1.11–6.22; *p* < 0.05) [43].

### 2.2. Targets of Autoantibodies in AHA

The A2, C1, and C2 domains were considered to be among the most active immunogenic domains of FVIII. Antibodies against FVIII are directed mainly towards A2 or C2 [44,45]. These antibodies bind to the 44-kD A2 and 72-kD domains of FVIII [46]. Certain clusters of differentiation (CD) 4+ T cell epitopes recognize the C2 domain of FVIII (2241–2290, 2191–2210, 2291–2330), resulting in the inhibition of the FVIII function [47]. The A3 domain of FVIII is a target of CD4+ T cells and leads to the inhibition of the factor [48]. The antibodies against the C2 domain are generally categorized as classical and nonclassical antibodies. Classical antibodies (epitopes A, AB, or B) do not allow FVIII to bind to platelet surfaces and vWf. Nonclassical antibodies (epitopes BC or C) constrain thrombin or FXa to activate FVIII, leading to an increased risk of bleeding and disruption of the hemostatic mechanism. Group BC inhibitors are the most common anti-C2 antibodies and are highly associated with AHA [49]. Kahle and colleagues conducted a study in 115 postpartum women with AHA, analyzing the antibodies against FVIII. The study concluded that the C1 domain of FVIII is the main target of the antibodies produced against the factor (in 78.2% of the patients with AHA) [44]. Lapalud et al. evaluated the autoantibodies produced against FVIII in a cohort of 36 patients with AHA (postpartum (*n* = 10), associated with malignancies (*n* = 12), and appeared with autoimmune disorders (*n* = 12)). The A1a1 domain of FVIII was the target of autoantibodies produced specifically in postpartum women with AHA (*p* < 0.01) [39]. Studies developed on postpartum women with AHA suggested that C1 and A1a1 of FVIII are the main targets of the autoantibodies produced. A2 inhibitors block the binding of FVIII to FX, while A3 inhibitors block the binding to FIXa, obstructing the formation of the Xase complex [26].

Recent evidence supports the concept that a more generalized failure of the peripheral control mechanisms of self-tolerance might be involved in AHA pathogenesis than an autoantibody offensive against specific domains of FVIII. Patients with AHA were found to be more frequently positive for antinuclear antibodies (ANAs), cytoplasmic autoantibodies, and anti-α-fodrin (AF) immunoglobulin A autoantibodies compared with the controls. The GTH-AH 01/2010 study presented that autoantibodies in patients with AHA are not only specific for FVIII but for other immune targets (ANA WITH human epithelial cell (HEp-2) immunofluorescence, AF nuclear, and cytoplasmic) (78% produced antibodies for any other target than FVIII compared with 46% of controls, OR 4.16, 1.98–8.39) [50].

### 2.3. Autoantibodies’ Mechanism of Action

**Figure 1:**
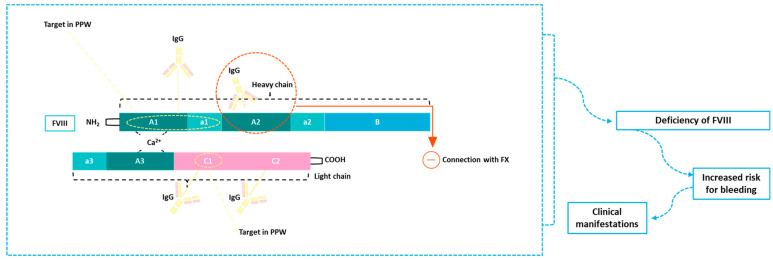
Main mechanism autoantibodies interacting with FVIII and contributing to the pathogenesis of AHA are presented. In the image, the structure of FVIII is presented. The A1a1 and C1 domains are the main targets of autoantibodies presented in PPW with AHA, as suggested by the published data. The interplay between IgG autoantibody and the A2 domain of FVIII leads to the inhibition of the connection of FVIII to FX. The C1 and C2 of the light chain of FVIII are targets of autoantibodies in patients with AHA. The deficiency of FVIII inducted by these mechanisms leads to increased risk of bleeding and final to clinical manifestations. PPW: postpartum women; FVIII: factor VIII; IgG: immunoglobulin G; FX: factor X.

FVIII autoantibodies can neutralize the factor’s activity via different pathways. The direct inhibition of FVIII by binding to functional regions or the enhancement of catabolism and clearance are the main mechanisms involved in the inactivation of FVIII in patients with AHA [51]. Autoantibodies in AHA are not specific only for FVIII [50]. The autoantibodies against FVIII can interplay with the factor via hydrolysis or proteolysis [52,53]. Increased hydrolytic activity has been associated with the presence of autoantibodies (patients with AHA) compared to alloantibodies (patient with HA and presence of inhibitory antibodies) (*p* = 0.03), as presented by the study of Wootla and colleagues [54]. In some AHA patients, FIX inhibitors can be found alone or in combination with the FVIII antibody. Specifically, in a cohort of 65 patients with AHA, the presence of hydrolyzing and activating FIX IgG antibodies leads to the activation of FIX in 25 patients (*p* < 0.05) [55]. In Figure 1, the main mechanism autoantibodies that interact with FVIII and contribute to the pathogenesis of AHA are presented.

## 3. Genetics in AHA

The major histocompatibility complex (MHC) represents a genetic component implicated in autoimmune disorders, with human leukocyte antigen (HLA) genes serving as a key constituent [56]. HLA genes exhibit a high degree of polymorphism and encode molecules crucial for presenting foreign antigens to the immune system. Although the precise mechanism governing MHC association with autoimmune pathology is still unclear, it is hypothesized that a breakdown in immunological tolerance to self-antigens, facilitated by the aberrant class II presentation of self or foreign peptides to autoreactive T lymphocytes, may play a significant role. Consequently, specific MHC class II alleles are presumed to dictate the targeting of particular autoantigens, thereby causing disease-specific associations [56]. Numerous investigations have showed significant correlations between distinct HLA alleles and autoimmune diseases like diabetes mellitus (DM) and rheumatoid arthritis [57,58]. Furthermore, studies have elucidated an increased susceptibility to inhibitor development in congenital severe hemophilia A linked to specific HLA alleles, including DQB1*06:02, DRB1*15:01, and DQA1*01:02 [59,60]. A comparative analysis of 57 individuals revealed that high-risk alleles in AHA patients, DRB1*16 and DQB1*05:02, were found to be low-risk alleles in congenital hemophilia A patients with inhibitors. Conversely, alleles associated with low risk in AHA, DRB1*15 and DQB1*06:02, emerged as high-risk alleles in hemophilia A inhibitor patients [56]. In a cohort of 49 AHA patients, HLA class I allele A*03:01 and class II allele DRB1*13:03 were found to be associated with AHA, thus reporting previously undescribed genetic factors for the disease [61]. The elucidation of such divergent allele profiles underscores the complexity of HLA involvement in autoimmune phenomena. In addition to HLA genes, the FVIII gene plays a crucial role in AHA development. Specific mutations compromising the primary structure of the FVIII protein, combined with distinct HLA profiles, may trigger aberrant antigen presentation and subsequent inhibitor formation in AHA patients [56,61]. It has proven that large deletions pose an extremely high risk (88%) for the development of inhibitors, and single-domain large deletions, nonsense mutations, intron 22 inversions, and small deletions have risk probabilities of 25%, 31%, 21%, and 16%, respectively [62]. Furthermore, novel mutations, such as c.8899G>A, have been implicated in AHA pathogenesis in two patients with the disease, further highlighting the genetic complexity underlying this disorder [63]. Nevertheless, the lack of an FVIII functional structure study and the presence of this mutation in two patients are two limitations of the previous study, confining the application of these data in clinical practice.

The encounter of variant allogeneic FVIII, presented on a suitable MHC background, emerges as a potential risk factor for inhibitor formation in AHA. This hypothesis is supported by findings of rare variants (c.6238G>A and c.3951C>G) in transfusion- and pregnancy-related AHA patients [64]. However, the lack of a functional investigation of the FVIII structure in patients with c.6238G>A and c.3951C>G variants limits the generalization of the concept that these mutations are ultimately pathogenetic for the onset of AHA. The identification of specific FVIII variants presented on patient HLA alleles underscores the role of MHC in modulating immune responses in AHA.

Among the factors implicated in the etiology of AHA, cytotoxic T lymphocyte antigen-4 (CTLA-4) emerges as a pivotal player in modulating T cell activation. CTLA-4 operates by binding to B7 receptor molecules, thereby delivering inhibitory signals crucial for dampening T cell activation. Recent investigations have shed light on CTLA-4’s involvement in autoimmune modulation, establishing it as a risk factor for inhibitor formation in inherited hemophilia A [65]. The *CTLA-4* gene, situated on chromosome 2q33 alongside other immunologically significant genes, harbors several genetic polymorphisms that have garnered attention. Notably, the CTLA-4 49 G allele has been observed at significantly higher frequencies in AHA patients compared to controls, particularly in female patients and those with underlying autoimmune diseases [66]. This allele’s association with AHA underscores the complex interplay between genetic variations and disease susceptibility. Regarding other polymorphic regions within the *CTLA-4* gene, research by Ueda et al. showcased a robust correlation between the CT60 A/G polymorphism and certain autoimmune conditions such as Graves’ disease, autoimmune hypothyroidism, and type 1 DM [67]. The variant CT60G has been linked to reduced levels of soluble CTLA-4 expression and influences the balance between transmembrane and soluble mRNA splice forms of the *CTLA-4* gene. Nevertheless, evidence supporting the correlation between this polymorphism and AHA remains inconclusive. Furthermore, no discrete link has been proved between the CTLA-4 promoter -318 C/T variant and AHA. The threonine to Alanine amino acid exchange encoded by this polymorphism alters the CTLA-4 receptor function, with the G/G genotype exhibiting diminished inhibitory effects on T cells compared to the *A/A* genotype. This observation suggests a potential deficiency in CTLA-4-dependent regulatory mechanisms, contributing to AHA pathogenesis. While the prevalence of AHA remains rare, the heightened expression of the CTLA-4 49 G allele in affected individuals underscores its role as one among several genetic factors predisposing to the disorder. In summary, CTLA-4 contributes to the development of AHA, with variations in the *CTLA-4* gene influencing disease susceptibility and manifestation [66].

Studies on autoimmune diseases have expanded to include natural killer (NK) cells, which regulate innate immune responses and eliminate cancer cells [68]. NK cell activity is governed by a complex network of inhibitory and activating cell surface receptors, such as killer cell immunoglobulin-like receptors (KIRs) and NKG2-D receptors. KIRs, encoded by the KIR locus (19q13.4), consist of 14 highly polymorphic genes encoding both inhibitory and activating receptors, in addition to two pseudogenes. These KIR receptors interact with HLA class I molecules, and their variations, known as KIR/HLA genotypes, have been linked to autoimmune diseases [69]. Similarly, NKG2-D, a type II integral membrane protein encoded by KLRK1 (12p13.2), recognizes ligands like MHC-class I polypeptide-related sequence A (MIC-A) and B (MIC-B) proteins on stressed cells [70]. The activation of NKG2-D receptors by these ligands triggers cell-mediated cytotoxicity, leading to the elimination of transformed and infected cells. Different haplotypes of KLRK1 have been linked with varying levels of NK cytotoxic activity, categorized as low (LNK) and high (HNK). Regarding *KLRK1* gene, rs1049174 polymorphism has been associated with AHA [61]. More precisely, a decreased occurrence was observed for the low-activity haplotype (LNK/LNK), while a higher prevalence of the heterozygous combination (HNK/LNK) and a complete absence of the high-activity haplotype (HNK/HNK) were noted. The absence of the homozygous HNK haplotype and the increased presence of the heterozygous combination among AHA patients were statistically significant. This distribution pattern suggests a predominance of the low-activity phenotype of NK cells in individuals with AHA. The functionality of NK cells is influenced by a delicate interplay of activating and inhibitory signals from cell surface receptors, which can modulate immune responses to varying degrees.

Moving forward, further studies regarding the genetic factors underlying AHA are essential for a comprehensive understanding of this complex disorder. Subsequent research should aim to expand the current knowledge by exploring additional candidate genes implicated in the immune response, alongside thorough analyses of the existing genetic associations. Large-scale studies with diverse and well-characterized cohorts of AHA patients are necessary to validate and refine the identified genetic associations. Moreover, leveraging advanced genomic technologies, such as next-generation sequencing (NGS), will enable researchers to uncover rare genetic variants and elucidate their contributions to AHA susceptibility. Studies investigating the genetic basis of AHA have focused on the study of the gene coding FVIII and genes associated with immune response towards FVIII. Mutations in the gene responsible for the synthesis of FVIII (F8) in CTLA4 and in HLAs have been addressed as pathogenetic for AHA by the published studies. In Table 1, the genes and polymorphisms associated with AHA are presented.

## 4. Epigenetics in AHA

Epigenetics refers to the mechanisms that alter DNA gene expression, without an impact on DNA sequence. Modifications involve DNA methylation, histone modifications, and non-coding RNAs (ncRNAs) [71]. Histone modifications include the well-studied acetylation, methylation, phosphorylation, and ubiquitylation, while O-linked-N-acetylglucosaminylation (GlcNAcylation), citrullination, crotonylation, and isomerization are the revolutionary discoveries of the histone modification field [72]. MicroRNAs (miRNAs), long non-coding RNAs (lncRNAs), and circular RNAs (circRNAs) constitute the main subtypes of the ncRNAs involved in the epigenetic alterations of the human genome [73]. Since 1983, it has been well known that epigenetics contribute to the onset and progress of human diseases [74].

Epigenetic factors could potentially contribute to the FVIII gene (mutated or not) and to the immunological mechanisms involved in the pathogenesis of AHA [75]. Environmental and epigenetic factors are possibly associated with the levels of FVIII. Biguzzi et al. presented that in patients with HA, the levels of VIII increase to 9IU/dl per decade (95% CI, 6–11) [76]. The methylation status of the gene that is coding the FVIII protein has been studied in patients with HA. Jamil and colleagues proposed that genetic alterations (Xq28) in the FVIII gene are interplaying with the methylation status of FVIII [77]. Nevertheless, another study with 80 hemophilic patients presented that the methylation patterns of FVIII gene promoters were similar to healthy controls [78]. In patients with HA but with a lack of mutations in FVIII, the increased expression of two ncRNAs, miR-374b-5p and miR-30c-5p, was associated with decreased levels of FVIII [79].

Only one study has been developed evaluating the potential role of epigenetics in the pathogenesis of AHA, studying the potential role of lncRNAs. Tigu et al. conducted a study in ten patients (with AH (*n* = 2) with mild HA (*n* = 3), with severe HA (*n* = 3), and healthy controls (*n* = 2)) and performed RNA sequencing analysis in the ncRNAs received from the peripheral blood. Performing bioinformatics analysis, they presented that in patients with AHA, an important number of altered transcriptomes were observed, suggesting the potential role of the ncRNAs in the pathogenesis of AHA [80]. While the study evaluated the same demographic characteristics, the small number of the participants is a limitation. The development of studies investigating the potential interplay between epigenetics, inflammation, and AHA is crucial to fully understand the pathogenetic mechanisms leading to the onset of the disorder More studies investigating the impact of epigenetic modifications on the levels and the structure of FVIII and on the development of autoantibodies towards FVIII, leading to the clinical onset of AHA, are needed to understand this complex disease.

## 5. Treatment Approaches in AHA

The management of patients with AHA is based on two main principles: one is the management of emergency and acute bleeding events presented in this group, and the other is immunosuppressive treatment. In this review, we present the management of the patient from the emergency department to bedside.

### 5.1. Management of Acute Bleeding Events

The guidelines of Tiede et al., published in 2020, constitute the foundation of the treatment approach of patients with AHA in everyday clinical practice [33]. The management of acute bleeding should be the first step in the therapeutic algorithm. Experienced staff should carry out venipuncture in these patients. Moreover, fasciotomy for the management of intramuscular bleeding is suggested to be avoided according to the published guidelines. Nevertheless, fasciotomy has been used in clinical practice in patients with severe life-threating intramuscular bleeding [81,82,83]. Early treatment is crucial for the prevention of compartment syndrome [84]. In the guidelines of Tiede et al., it is recommended that hemostatic treatment should be administered in AHA patients with bleeding, which is clinically relevant. It is underlined that the titer of inhibitors and FVII residual activity should not influence clinical decision, as inhibitor level and FVIII residual activity is not correlated with phenotype in many patients [33].

**Figure 2:**
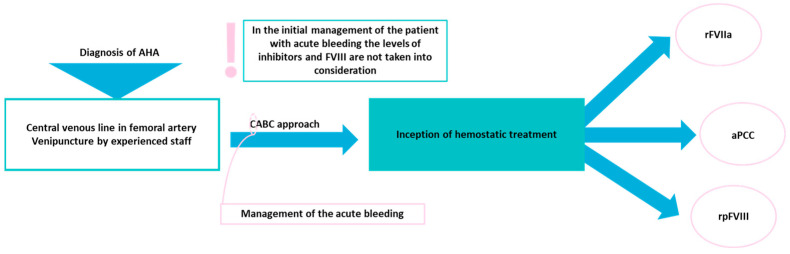
Management of acute bleeding events in patients with AHA. AHA: acquired hemophilia A; FVIII: factor VIII; CABC: circulation airway breathing circulation; rFVIIa: recombinant factor VIIa; aPCCs: activated prothrombin complex concentrates; rpFVIII: recombinant porcine FVIII.

Hemostatic agents that can be used include recombinant FVIIa (rFVIIa), activated prothrombin complex concentrates (aPCCs), and recombinant porcine FVIII (rpFVIII) [23,85]. Data from retrospective and prospective studies support that the clinical efficacy of the three agents in the management of AHA is similar [86,87]. In a systematic review of 32 studies, including 671 patients treated with rFVIIa, it was found that the treatment efficacy was >90% (data from five studies examining treatment response) [88]. As suggested, in the initial treatment with rFVIIa, bolus injections of 90 μg/kg every 2–3 h should be used until hemostasis is achieved [33]. In the study of Mingot-Castellano et al., aPCCs as first-line treatment ended the bleeding in 13 out of 14 (92.9%) AHA patients, while as a second-line treatment, it stopped the bleeding in all the patients reported [89]. aPCCs are also used as a prophylactic agent for bleeding events in patients with acquired hemophilia and inhibitors [90]. In the guidelines of Tiede et al., bolus injections of aPCCs between 50 and 100 U/kg (starting doses) every 8–12 h, up to a maximum of 200 U/kg/day, are recommended [33]. Furthermore, the administration of aPCCs is contradicted in patients with AHA and signs of disseminated intravascular coagulation [33]. When rpFVIII is chosen as the initial treatment, a dose of 200 U/kg, followed by additional doses to sustain the levels of FVIII > 50% through the treatment, is used [33]. Studies are suggesting that, during therapy with rpFVIII, the activity of FVIII should be monitored [33,91]. Furthermore, de novo anti-rpFVIII inhibitors can be developed in these patients, leading to discontinuation of the treatment [92,associated with malignan^87^]. Treatment response to hemostatic agents should be evaluated clinically, and a treatment switch might be implemented if the chosen hemostatic agent is found to be ineffective [93]. Initial treatment with human FVIII should be limited only to cases when access to the above three agents is not available and in patients with low-titer inhibitors (<5 BU) [33]. Plasma-deprived FVIIa/FX has been also evaluated in patients with AHA, with favorable outcomes without an increase in the thrombotic risk [94]. Emicizumab, a monoclonal antibody restoring the function of missing FVIII by connecting FIXa and FX, has been used off-label in AHA [95,96,97,98,99]. Figure 2 presents the initial clinical administration of the patient with AHA in the emergency room department.

### 5.2. Immunosuppressive Treatment

The administration of immunosuppressive agents is suggested in patients with AHA, but an individualized approach should be applied to frail patients who might be at a great risk of mortality related to immunosuppressive treatment (IST) [33]. Levels of FVIII at baseline and inhibitor titers can be helpful in a personalized approach to the treatment of AHA [100].

First-line therapy with corticosteroids alone should be considered in patients with FVIII ≥ 1 IU/dL and inhibitor titer ≤ 20 BU, while in those with FVIII < 1 IU/dL or inhibitor titer > 20 BU, corticosteroids with rituximab or a cytotoxic agent should be used as first-line treatment [33]. Prednisone or prednisolone (1 mg/kg/day per os (PO)) can be used for 4–6 weeks. A maximum of four treatment cycles of rituximab (375 mg/m^2^ weekly) can be administered in those patients based on the current guidelines. In a recently published retrospective study, 80 participants who received corticosteroids combined with rituximab IST had a 93.3% complete remission rate [101]. Rituximab has been used as an immunosuppressant in various benign and malignant hematological disorders [102,103,104] as cytotoxic agents’ cyclophosphamide (PO, 1.5–2 mg/kg/day, for a maximum of 6 weeks) or mycophenolate mofetil (1 g/day for 1 week, and after 2 g/day). In a randomized controlled study comparing corticosteroids plus rituximab with corticosteroids plus cyclophosphamide in patients with AHA, similar efficacy and safety were reported between the two drug combinations [105]. During treatment with cytotoxic agents, patients should be watched closely for the development of cytopenias or infections [106,107,108,109,110].

## 6. Conclusions

AHA is a disorder that can be life threating for patients, with an estimated mortality rate of 7.9% to 22% [111,112,113,114]. AHA can be attributed to hematological malignancies, solid tumors, autoimmune disorders, pregnancy, drugs, and other factors, or it can be presented as an individual clinical disorder [17]. The pathogenesis of AHA is based on the immunological inhibition of FVIII with the production of autoantibodies, mainly IgG. The activation of immunity towards FVIII is associated with genetic factors as well. Genetic alterations mainly in the *F8* gene, and in the CTLA-4, HLA, and KLRK1 immune regulatory genes, are associated with AHA. Epigenetic modifications have been associated with the onset of AHA; nevertheless, only one study has been published investigating the potential role of lcRNAs in the modulation of FVIII expression. Further research in the field of AHA is needed to understand the complex severity of this disease.

Recently, efforts have been made in order to understand the genetic modifiers affecting the clinical phenotypes of patients with hemoglobinopathies [115,116]. These works are inspirational to better understand patients with congenital HA and HB or AHA. To this direction, both whole-genome and next-generation sequence analysis in AHA are essential to understand the pathogenesis of this complex clinical entity. Furthermore, given that very few data are available toward the contribution of epigenetic alterations in the pathogenesis of AHA, more research is needed to provide insights in this field. A question that has arisen is whether innate immune system (Toll-like receptors, complement system) dysregulation is implicated in the immune dysfunction observed in patients with AHA [117,118]. The long-term follow-up of patients with acquired bleeding disorders is vital for the identification of long-term complications such as disease relapse, increased burden of CVD, and thromboembolic events [119]. Multicenter collaboration could assist given the rarity of AHA.

## Figures and Tables

**Table 1:** Table of genes, polymorphisms, and association with acquired hemophilia.

Gene	Polymorphisms/Alleles	Comments	References
*CTLA-4*	49 A/G	AHA Risk Factor	Pavlova et al., 2008 [66]
	60 G/A	AD Risk Factor	Ueda et al., 2003 [67]
	-318 C/T	AD Risk Factor	Ueda et al., 2003 [67]
*FVIII*	c.8899G>A	AHA Risk Factor	Hwang et al., 2011 [63]
	c.3951C>G	AHA Risk Factor	Tiede et al., 2010 [64]
	c.6238G>A	AHA Risk Factor	Tiede et al., 2010 [64]
*KLRK1*	rs1049174	AHA Risk Factor	Pardos-Gea et al., 2023 [61]
*HLA*	A*03:01	AHA Risk Factor	Pardos-Gea et al., 2023 [61]
	DRB1*13:03	AHA Risk Factor	Pardos-Gea et al., 2023 [61]
	DRB1*16	AHA Risk Factor	Pavlova et al., 2010 [56]
	DQB1*05:02	AHA Risk Factor	Pavlova et al., 2010 [56]
	DRB1*15	AHA Low Risk Factor	Pavlova et al., 2010 [56]
	DQB1*06:02	AHA Low Risk Factor	Pavlova et al., 2010 [56]

* D: autoimmune diseases, AHA: acquired hemophilia A.

## Abbreviations

AF	α-fodrin
AHA	Acquired hemophilia A
aHR	Adjusted hazard ratio
ANAs	Antinuclear antibodies
aPCCs	Activated prothrombin complex concentrates
BUs	Bethesda units
CD	Clusters of differentiation
CI	Confidence interval
CIRCRNAS	Circular RNAs
CTLA-4	Cytotoxic T lymphocyte antigen-4
CVD	Cardiovascular disease
DM	Diabetes mellitus
FIX	Factor IX
FVIII	Factor VIII
FX	Factor X
GlcNAcylation	O-linked-N-acetylglucosaminylation
HA	Hemophilia A
HB	Hemophilia B
HLA	Human leukocyte antigen
HEp-2	Human epithelial cell-2
Ig	Immunoglobulin
KIRs	Killer cell immunoglobulin-like receptors
lncRNAs	Long-coding RNAs
MHC	Major histocompatibility complex
miRNAs	microRNAs
NGS	Next-generation sequencing
NK	Natural killer
PO	Per os
rFVIIa	Recombinant FVIIa
rpfviii	Recombinant porcine FVIII
vWf	Von Willebrand Factor
